# Association of the fibrosis-4 index with early-stage cardiovascular-kidney-metabolic syndrome in a longitudinal community-based cohort

**DOI:** 10.3389/fpubh.2026.1793972

**Published:** 2026-03-18

**Authors:** Jing He, Cheng Zhang, Hui Luo, Mengjie Sun, Ruilei Hu, Xueting Wang, Yanlang Yang, Shu Xiao, Zhonghui Chen, Xianhong Yin

**Affiliations:** 1 Department of Nephrology, The Affiliated Xuancheng Hospital of Wannan Medical College, Xuancheng, China; 2 Anhui Proof of Concept Center, Hefei, China; 3 Department of Nephrology, The First Affiliated Hospital of Wannan Medical College, Yijishan Hospital, Wuhu, China; 4 Department of Central Laboratory, The Affiliated Xuancheng Hospital of Wannan Medical College, Xuancheng, China

**Keywords:** cardiovascular-kidney-metabolic syndrome, FIB-4, longitudinal cohort, non-invasive biomarker, risk factors

## Abstract

**Background:**

Cardiovascular-kidney-metabolic (CKM) syndrome, a systemic disorder affecting approximately 25% of U.S. adults, accounts for over 75% of healthcare costs. As a central metabolic regulator, the liver may play a pivotal role in the development of CKM. Although FIB-4, a non-invasive liver injury marker, correlates with cardiovascular disease, its value as an independent predictor of incident CKM syndrome remains unclear. We therefore investigated this association using baseline FIB-4 data.

**Methods:**

We conducted a retrospective cohort study using data from the Health and Aging Trends Study in Anhui, China, including 1,633 adults aged ≥20 years. Baseline FIB-4 was log-transformed and analyzed continuously and in tertiles. Logistic regression models estimated odds ratios (ORs) for incident CKM syndrome at 1, 3, and 5 years, with sequential adjustment for demographics, lifestyle factors, comorbidities, and metabolic variables. Restricted cubic spline analysis explored non-linear associations. Missing data were addressed via multiple imputation.

**Results:**

Among 1,633 participants, 675, 798, and 910 developed CKM syndrome at 1, 3, and 5 years, respectively. Higher baseline FIB-4 was consistently associated with greater odds of incident CKM syndrome. In fully adjusted models, each one-log-unit increase in FIB-4 was associated with ORs of 1.32 (95% CI, 1.03–1.69) at 1 year, 1.31 (1.02–1.68) at 3 years, and showed a borderline association at 5 years (1.27, 0.99–1.63).Compared with the lowest tertile, the highest FIB-4 tertile showed significantly elevated risk: ORs of 1.50 (1.07–2.09), 1.75 (1.25–2.46), and 1.45 (1.03–2.05), with significant linear trends (*P* < 0.05). Subgroup analyses showed consistent associations with no significant interactions.

**Conclusion:**

Elevated FIB-4 index was independently associated with a higher risk of incident CKM syndrome in a dose-dependent manner, suggesting liver injury may represent a potential target for early prevention.

## 1 Introduction

Recognizing the increasingly close interrelationships among metabolic disease, cardiovascular disease (CVD), and chronic kidney disease (CKD), the American Heart Association (AHA) has recently proposed and established a consensus definition for the concept of cardiovascular-kidney-metabolic (CKM) syndrome as a systemic disorder that is characterized by multidirectional interactions among these systems, significantly elevating the risk of adverse cardiovascular outcomes and multi-organ dysfunction (1). Substantial epidemiologic evidence shows that CVD risk rises progressively across the CKM spectrum (in the absence of risk factors) to stage 4 (with confirmed CVD), and denotes potential adverse outcomes and the leading cause of death (2, 3). From historical data, approximately 25% of U.S. adults simultaneously experienced CKM and were responsible for over 75% of total American healthcare costs (4–6). Given that CVD constitutes the primary burden of CKM, a comprehensive, integrated management strategy that targets metabolic, renal, and cardiovascular health is essential. This requires moving beyond isolated interventions to holistically address obesity, diabetes, and CKD, thereby mitigating disease development and progression.

CKM is characterized by multiorgan dysfunction that is driven by shared metabolic disturbances that include insulin resistance, oxidative stress, and chronic inflammation (1, 7). The liver acts as a central metabolic regulator and is a primary site of injury, with hepatic damage initially serving as a mediating pathway that amplifies metabolic dysfunction, then transitioning into an upstream driver once fibrosis is established (8). Such injury disrupts inter-organ crosstalk along the hepatic-cardiac-renal axis, propagating pathological signals that accelerate disease progression across multiple organ systems (8–10). This hepatic injury is exemplified by metabolic dysfunction-associated steatohepatitis (MASH), which combines with hepatic lobular inflammation and variable severities of hepatic fibrosis, and is often driven by modifiable risk factors such as obesity, metabolic dysregulation, unhealthy dietary patterns, and sedentary behavior (11). The resulting hepatic pathology not only increases the risk of progressive liver disease, but also initiates and sustains systemic consequences that include elevated cardiovascular risk, CKD, and extrahepatic malignancies (8). Thus, liver injury serves as a critical pathological nexus in CKM syndrome, where localized hepatic damage translates into systemic metabolic and inflammatory dysregulation and creates a self-perpetuating cycle of multiorgan dysfunction (8, 11, 12). Consequently, elucidating the mechanistic pathways by which hepatic injury contributes to CKM syndrome is essential for the development of stratified diagnostic approaches and targeted therapeutic interventions that address this central node in disease pathogenesis.

The fibrosis-4 (FIB-4) index is a widely used non-invasive marker of hepatic fibrosis and liver injury, and has been increasingly recognized in multiple studies for its association with hepatic disease (13–15). As a composite index derived from age, liver enzyme concentrations [alanine aminotransferase (ALT) and aspartate aminotransferase (AST)], and platelet count, FIB-4 improves the detection of subclinical liver injury and fibrosis, which are often linked to metabolic dysfunction and insulin resistance. Emerging research suggests that elevated FIB-4 scores are significantly correlated with an increased incidence of CVD, independent of traditional risk factors, potentially due to shared pathways that involve chronic inflammation, oxidative stress, and endothelial dysfunction mediated by liver injury (16–18). However, it remains to be clearly established whether this relationship holds true within a population with CKM syndrome, a condition that is characterized by multiorgan metabolic dysregulation.

Herein, we investigated the relationship between the FIB-4 index and incidence of early-stage CKM syndrome. We posit that elucidation of the independent predictive value of liver injury markers for CKM syndrome development will offer crucial evidence for the formulation of targeted preventive strategies in the early stages, while also potentially alleviating the escalating cardiovascular burden linked to CKM progression.

## 2 Methods

### 2.1 Study population

**Figure 1:**
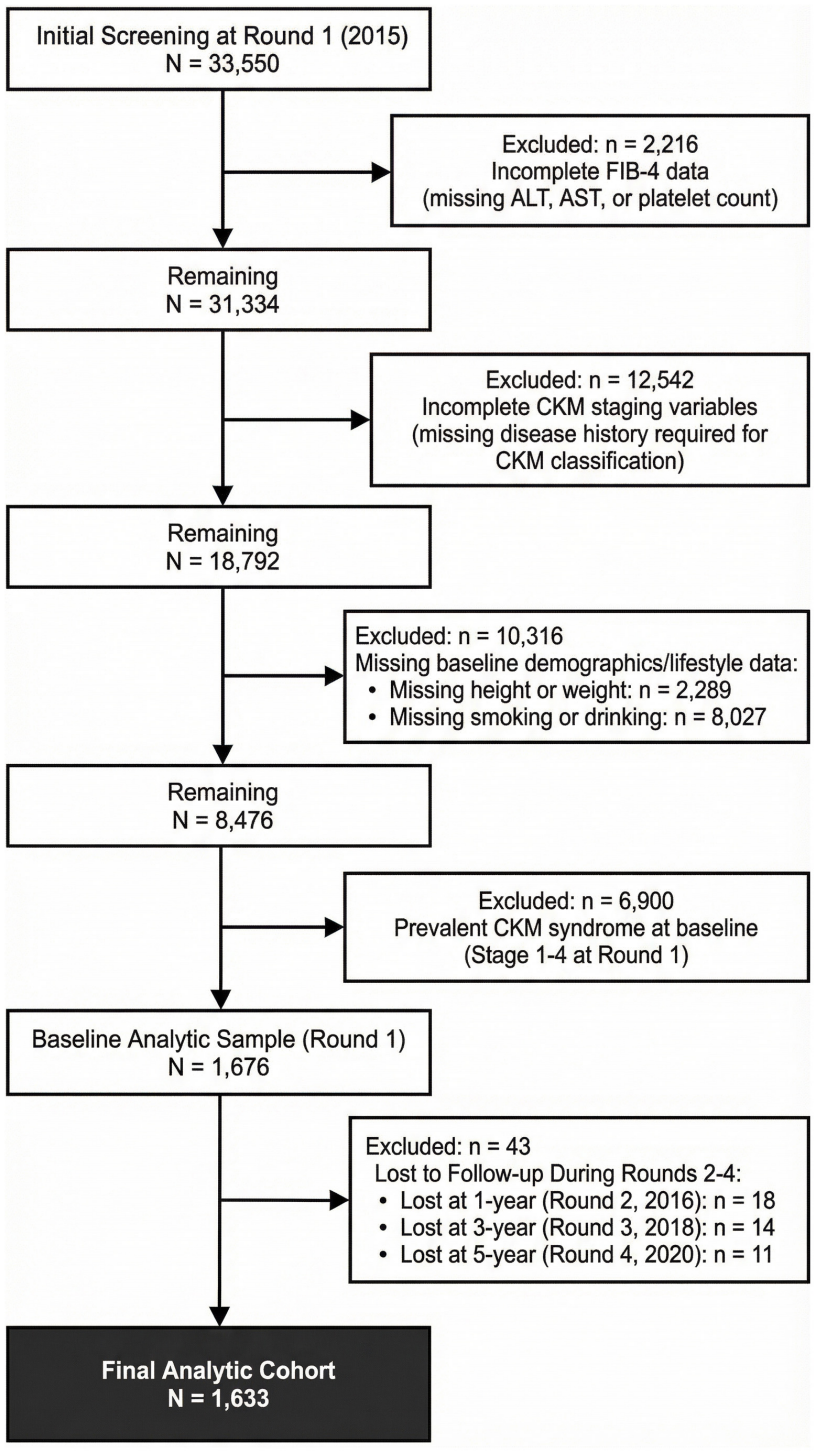
Flow diagram of participant selection.

This was a retrospective cohort study with data collected from an urban community as part of the Health and Aging Trends Study, a longitudinal survey of healthcare security beneficiaries aged ≥20 years and residing in Anhui, China. Blood samples were collected annually from 2015 onward (Round 1). In this analysis, we used data from five rounds of collection [2015 (Round 1), 2016 (Round 2), 2018 (Round 3), and 2020 (Round 4)]. At Round 1, a total of 1,676 individuals were recruited, and of the all participants aged 20 years and above, we excluded 31,874 participants for the following reasons: (1) lack of demographic data at baseline, (2) lack of clinical history data at baseline, (3) inability to calculate FIB-4, and (4) inability to accurately diagnose CKM syndrome stage. During the follow-up period, 43 participants lacked complete clinical characteristics and/or withdrew from the study. Finally, 1,633 individuals were enrolled and analyzed in this longitudinal setting (Figure 1). This study used de-identified human data without bodily harm, sensitive information, or commercial interests, complying with the Declaration of Helsinki. The Xuancheng People's Hospital Ethics Committee granted ethical exemption.

### 2.2 Covariates

Covariates collected at baseline through a self-reported questionnaire included demographic and clinical characteristics or clinical history. The demographic data included age, sex, marital status, and educational status; lifestyles included smoking and drinking histories. Physical examinations (e.g., height, weight, and blood pressure measurement) were performed by trained physicians. Body mass index (BMI, weight in kilograms divided by height in meters squared) was categorized into normal, overweight, and obese. The clinical characteristics comprised self-reported CVD, hypertension, diabetes and hepatic disorders. Self-reported CVD included cerebral infarction, stroke, cerebral hemorrhage, coronary heart disease, myocardial infarction, and heart failure. Fasting blood samples were collected for hematological data that included ALT, AST, total bilirubin (TBil), direct bilirubin (DBil), creatinine (Cr), blood urea nitrogen (BUN), uric acid (UA), high-density lipoprotein (HDL), low-density lipoprotein (LDL), fasting blood glucose (FBG) and triglyceride (TG) levels. All laboratory measurements were conducted in a certified laboratory following standardized protocols.

### 2.3 Measurement of the FIB-4 index

The fibrosis-4 index (FIB-4) served as the exposure variable in this study. We calculated FIB-4 using ALT, AST, PLT, and age according to the following formula (13):


$$FIB-4=\frac{Age\;(years)\times AST\;(U/L)}{Platelet\;count\;(10^{9}/L)\;\times \sqrt{ALT\;(U/L)}}$$ (1)

### 2.4 Definitions of CKM syndrome

According to AHA statements, CKM syndrome is a multiorgan disorder defined by pathophysiological interactions among CVD, CKD, and metabolic relative risk factors (1). We classified the participants into stages 0–4 as follows: Stage 0, individuals without overweight/obesity, metabolic risk factors; Stage 1, individuals with overweight/obesity, abdominal obesity, or dysfunctional adipose tissue, without the presence of other metabolic risk factors or CKD; Stage 2, individuals with metabolic risk factors or CKD; Stage 3, individuals with excess/dysfunctional adiposity, other metabolic risk factors, or CKD in subclinical CVD or subclinical heat failure; Stage 4, individuals with clinical CVD. Subclinical CVD was defined as predicted 10-year ASCVD risk ≥20% [calculated using the China-PAR equations (19), consistent with the AHA definition of high cardiovascular risk] or very high-risk CKD stage. Participants classified as “Stage 0” were defined as CKM negative, whereas those in “Stages 1–4” were defined as CKM positive.

### 2.5 Statistical analysis

Continuous variables are expressed as mean ± standard deviation or median (interquartile range) according to their distribution, and categorical variables as counts (percentages). FIB-4 was log-transformed to approximate a normal distribution (Supplementary Figure 1). Differences in baseline variables across log-transformed FIB-4 tertiles were evaluated with one-way ANOVA or Kruskal–Wallis tests for continuous variables and χ^2^ tests for categorical variables. Tertile boundaries for baseline FIB-4 were 0.112–0.811 (T1), 0.812–1.340 (T2), and 1.341–12.000 (T3) on the original scale.

We executed logistic regression to estimate odds ratios (ORs) and 95% confidence intervals (CIs) for the associations between baseline log-transformed FIB-4 values (both per one log-unit increment and across tertiles) and diagnosis of CKM syndrome at one-, three-, and 5-year follow-up. Three sequential models were constructed in complete-case data: model 1 was the crude model; model 2 was adjusted for age, sex, smoking status, alcohol consumption, and comorbidities; and model 3 was additionally adjusted for BMI, renal function, blood pressure, hepatic function, lipid profile, and medication use. We selected potential confounders *a priori* on the basis of the existing literature and a directed acyclic graph (DAG) constructed to identify the minimal sufficient adjustment set for estimating the effect of FIB-4 on incident CKM syndrome (Supplementary Figure 2). Tests for linear trends were conducted by entering the median log-transformed FIB-4 value for each tertile as a continuous variable into the models.

Restricted cubic spline (RCS) logistic regression was employed to characterize the shape of the association between the FIB-4 index and the risk of CKM syndrome. Three knots were placed at the 10th, 50th, and 90th percentiles of log-FIB4, and the 33rd percentile value was taken as the reference. A fully-adjusted logistic model was constructed, and non-linearity was evaluated with the likelihood-ratio test in which we compared the model containing only the linear term with the model that also included the spline terms. To obtain interpretable ORs across the continuous exposure range, we computed pairwise contrasts between every value on the observed log-FIB4 distribution and the reference value while keeping all other covariates fixed at the reference levels.

We performed sensitivity analyses by fitting the fully adjusted model (model 3) separately within the strata of sex, age (< 65 vs. ≥65 years), tobacco smoking (never vs. ever), and alcohol consumption (none, less frequent, or frequent). Interaction effects were evaluated by entering cross-product terms between the continuous log-transformed FIB-4 value and each grouping variable into the corresponding model; the *P*-value for interaction was derived from the Wald test of the product terms. The proportions of missing data for crucial variables are detailed in Supplementary Table 1. In order to examine the impact of missing data on results, 20 imputed datasets were created and logistic models that regressed CKM syndrome occurrence on log-transformed FIB-4 were fitted in each. Estimates were then pooled across imputations by Rubin's rules. To assess the incremental predictive value of FIB-4, two models were constructed with one model including log-transformed FIB-4 alongside covariates from model 3 and another model excluding FIB-4. Receiver operating characteristic (ROC) curve analysis was performed to compute area under the curve (AUC) values with 95% CIs for both models, followed by visualization of ROC curves.

All analyses were conducted using R 4.4.1 and were two-tailed, with *P* < 0.05 considered to be statistically significant.

## 3 Results

### 3.1 Baseline characteristics of study participants

Participants in the highest tertile (T3) were markedly older (median age 54 vs. 32 years), more likely to be male (52.7 vs. 28.5%), and displayed a higher proportion of tobacco smoking and frequent alcohol consumption than those in the lowest log-FIB-4 tertile (T1). They also exhibited higher levels of diastolic blood pressure, FBG, Cr, BUN, TGs, total cholesterol, HDL, gamma-glutamyl transferase, AST, ALT, and UA; and lower platelet, red blood cell, and white blood cell counts (all *P* values < 0.05), whereas BMI, LDL, and hemoglobin remained comparable across groups (Table 1).

**Table 1:** Baseline characteristics of the study population stratified by fibrosis-4 index tertiles (*N* = 1,633).

**Variable**	**Tertile 1 (*n* = 708)**	**Tertile 2 (*n* = 549)**	**Tertile 3 (*n* = 376)**	***P*-value**
**Sex**, ***n*** **(%)**				< 0.001
Female	506 (71.5)	340 (61.9)	178 (47.3)	
Male	202 (28.5)	209 (38.1)	198 (52.7)	
Age [years, median (IQR)]	32 (28, 36)	41 (35, 47)	54 (45, 65)	< 0.001
≥65 years, *n* (%)	0 (0.0)	11 (2.0)	102 (27.1)	< 0.001
< 65 years, *n* (%)	708 (100.0)	538 (98.0)	274 (72.9)	
**Tobacco smoking**, ***n*** **(%)**				< 0.001
Non-smoker	603 (91.6)	448 (86.5)	283 (82.3)	
Smoker	55 (8.4)	70 (13.5)	61 (17.7)	
**Alcohol drinking**, ***n*** **(%)**				0.005
Non-drinker	579 (88.1)	448 (86.2)	283 (82.3)	
Less frequent	46 (7.0)	56 (10.8)	48 (14.0)	
Frequent	32 (4.9)	16 (3.1)	13 (3.8)	
Height (years, mean ± SD)	163.14 ± 7.65	163.56 ± 8.00	163.12 ± 7.74	0.627
Weight (kg, mean ± SD)	54.93 ± 6.85	55.56 ± 6.72	55.25 ± 6.64	0.329
BMI (kg/m^2^, mean ± SD)	20.78 (19.48, 21.86)	20.93 (19.79, 21.91)	21.01 (19.62, 21.91)	0.261
SBP [mmHg, median (IQR)]	70 (65, 70)	70 (65, 70)	70 (65, 70)	0.214
DBP [mmHg, median (IQR)]	100 (100, 110)	100 (100, 110)	110 (100, 115)	< 0.001
FG (mmol/L, mean ± SD)	5.07 ± 0.46	5.10 ± 0.47	5.22 ± 0.57	< 0.001
CR [μmol/L, median (IQR)]	55.60 (48.40, 66.90)	60.30 (52.80, 72.57)	65.10 (54.90, 78.70)	< 0.001
BUN (mmol/L, mean ± SD)	4.86 ± 1.32	5.12 ± 1.38	5.70 ± 1.52	< 0.001
TG [mmol/L, median (IQR)]	0.90 (0.70, 1.16)	0.93 (0.74, 1.21)	0.96 (0.77, 1.22)	0.010
TC [mmol/L, median (IQR)]	4.07 (3.64, 4.51)	4.20 (3.74, 4.62)	4.25 (3.82, 4.68)	< 0.001
LDL-C (mmol/L, mean ± SD)	2.02 ± 0.59	2.03 ± 0.58	2.06 ± 0.55	0.618
HDL-C (mmol/L, median (IQR)]	1.54 (1.36, 1.79)	1.63 (1.44, 1.86)	1.64 (1.42, 1.93)	< 0.001
TP (g/L, mean ± SD)	74.51 ± 3.95	73.94 ± 3.72	73.29 ± 4.15	< 0.001
ALB (g/L, mean ± SD)	47.38 ± 3.15	46.86 ± 2.99	46.19 ± 3.14	< 0.001
GLO (g/L, mean ± SD)	27.13 ± 3.45	27.09 ± 3.51	27.10 ± 3.70	0.973
HGB (g/L, mean ± SD)	133.83 ± 15.56	134.50 ± 14.96	134.35 ± 12.88	0.707
RBC ( × 10^12^/L, mean ± SD)	4.51 ± 0.45	4.47 ± 0.43	4.37 ± 0.45	< 0.001
PLT ( × 10^12^/L, mean ± SD)	224.52 ± 45.19	181.23 ± 39.48	134.69 ± 39.77	< 0.001
WBC ( × 10^12^/L, mean ± SD)	6.16 ± 1.45	5.66 ± 1.50	5.47 ± 1.56	< 0.001
GGT [U/L, median (IQR)]	12.00 (8.00, 17.00)	11.00 (8.00, 16.00)	14.00 (10.00, 20.00)	< 0.001
AST [U/L, median (IQR)]	15.00 (13.00, 18.00)	17.00 (15.00, 20.00)	20.00 (17.00, 24.00)	< 0.001
ALT [U/L, median (IQR)]	14.00 (11.00, 20.00)	14.00 (11.00, 20.00)	17.00 (12.00, 22.25)	< 0.001
UA (μmol/L, mean ± SD)	278.46 ± 65.96	281.56 ± 68.57	308.94 ± 73.54	< 0.001

SD, standard deviation; IQR, interquartile range; BMI: body mass index; SBP: systolic blood pressure; DBP: diastolic blood pressure; FG: fasting glucose; CR: serum creatinine; BUN: blood urea nitrogen; TG: triglycerides; TC: total cholesterol; LDL-C: low-density lipoprotein cholesterol; HDL-C: high-density lipoprotein cholesterol; TP: total protein; ALB: albumin; GLO: globulin; RBC: red blood cell count; HGB: hemoglobin; PLT: platelet count; WBC: white blood cell count; GGT: gamma-glutamyl transferase; AST: aspartate aminotransferase; ALT: alanine aminotransferase; UA: uric acid.

### 3.2 Association between FIB-4 and the risk of CKM syndrome

**Figure 2:**
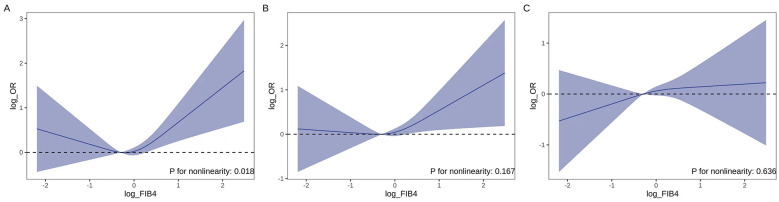
**(A–C)** Dose-response association between baseline log-transformed FIB-4 and the risk of CKM syndrome at 1, 2, and 5 years of follow-up as estimated using RCS logistic regression (with three knots at the 10th, 50th, and 90th percentiles). Solid lines represent adjusted ORs; shaded bands indicate 95% CIs. A likelihood-ratio test suggested significant non-linearity for 1-year risk (*P* = 0.018). FIB-4, fibrosis-4; CKM, cardiovascular-kidney-metabolic; RCS, restricted cubic spline; OR, odds ratio; CI, confidence interval.

A total of 1,633 participants from baseline encompassed the analysis; 675, 798, and 910 individuals met the diagnostic criteria of CKM syndrome at one-, three-, and 5-year follow-up, respectively. Higher FIB-4 was consistently associated with increased odds of CKM syndrome at 1- and 3-year follow-up. In fully adjusted analyses (model 3), each one-log-unit increment in FIB-4 conferred ORs of 1.32 (95% CI, 1.03–1.69) at 1 year and 1.31 (1.02–1.68) at 3 years. At 5 years, the association remained directionally consistent but reached borderline statistical significance (OR 1.27, 0.99–1.63). When log-FIB-4 was examined in tertiles, participants in the highest tertile exhibited a significantly higher risk for CKM than individuals in the lowest tertile across all timepoints: one-year OR, 1.50 (1.07–2.09); 3-year OR, 1.75 (1.25–2.46), and 5-year OR, 1.45 (1.03–2.05), with *P* values for the linear trend test of 0.034, 0.002, and 0.025, respectively (Table 2). We then further explored the shape of the association. Figure 2 shows that a non-linear relationship was evident only for 1-year risk (*P* = 0.018), whereas a linear model appeared to be more appropriate for the 3- and 5-year analyses.

**Table 2:** Associations of baseline FIB-4 level with 1-, 3-, and 5-year risk of CKM syndrome across different modeling strategies.

**Log-transformed FIB-4**	**Model 1**			**Model 2**			**Model 3**		
	**OR (95% CI)**	***P*** **-value**	***P*** **for trend**	**OR (95% CI)**	***P*** **-value**	***P*** **for trend**	**OR (95% CI)**	***P*** **-value**	***P*** **for trend**
**One-year risk of CKM syndrome**	
Per one log-unit	1.62 (1.36, 1.94)	< 0.001	—	1.30 (1.06, 1.61)	0.013	—	1.32 (1.03, 1.69)	0.026	—
Tertile 1	Reference		< 0.001	Reference		0.011	Reference		0.034
Tertile 2	1.06 (0.85, 1.34)	0.594		1.06 (0.84, 1.35)	0.606		1.01 (0.77, 1.34)	0.930	
Tertile 3	1.92 (1.49, 2.48)	< 0.001		1.52 (1.13, 2.03)	0.005		1.50 (1.07, 2.09)	0.018	
**Three-year risk of CKM syndrome**			
Per one log-unit	1.69 (1.42, 2.02)	< 0.001	—	1.33 (1.08, 1.64)	0.007	—	1.31 (1.02, 1.68)	0.031	—
Tertile 1	Reference		< 0.001	Reference		0.001	Reference		0.002
Tertile 2	1.23 (0.98, 1.54)	0.071		1.19 (0.94, 1.50)	0.150		1.14 (0.87, 1.50)	0.342	
Tertile 3	2.15 (1.67, 2.79)	< 0.001		1.68 (1.25, 2.25)	0.001		1.75 (1.25, 2.46)	0.001	
**Five-year risk of CKM syndrome**			
Per one log-unit	1.69 (1.41, 2.03)	< 0.001	—	1.32 (1.07, 1.64)	0.010	—	1.27 (0.99, 1.63)	0.061	—
Tertile 1	Reference		< 0.001	Reference		0.006	Reference		0.025
Tertile 2	1.35 (1.08, 1.70)	0.008		1.24 (0.98, 1.58)	0.071		1.23 (0.94, 1.62)	0.140	
Tertile 3	2.02 (1.56, 2.62)	< 0.001		1.48 (1.10, 2.00)	0.010		1.45 (1.03, 2.05)	0.032	

FIB4, fibrosis-4 index; CKM, cardiovascular-kidney-metabolic; OR, odds ratio; CI, confidence interval. Model 1 is the crude model. Model 2 adjusts age, sex, smoking, alcohol drinking, and comorbidity. Model 3 adjusts age, sex, smoking, alcohol drinking, comorbidity, body mass index, renal function, blood pressure, hepatitis, lipid profile, and drug use.

### 3.3 Subgroup analyses

To further examine the relationship between FIB-4 and the incidence of CKM, we enacted subgroup and interaction analyses across different age groups, sex, and smoking and alcohol drinking statuses. The association between a one-log-unit rise in FIB-4 and CKM syndrome was largely consistent across subgroups (Table 3).

**Table 3:** Subgroup analyses of associations of FIB-4 increase per one log-unit with 1-, 3-, and 5-year risk of CKM syndrome.

**Group**	**1-year risk**			**3-year risk**			**5-year risk**		
	**OR (95% CI)**	***P*** **-value**	*P*_interaction_	**OR (95% CI)**	***P*** **-value**	*P*_interaction_	**OR (95% CI)**	***P*** **-value**	*P*_interaction_
**Sex**	
Male	1.45 (1.06, 1.99)	0.022	0.365	1.44 (1.05, 1.98)	0.023	0.334	1.51 (1.11, 2.08)	0.010	0.071
Female	1.18 (0.83, 1.67)	0.359		1.15 (0.81, 1.65)	0.438		0.98 (0.67, 1.43)	0.915	
**Age**	
≥65	1.30 (1.01, 1.68)	0.042	0.604	1.37 (1.06, 1.76)	0.016	0.153	1.32 (1.02, 1.70)	0.035	0.164
< 65	1.66 (0.62, 4.63)	0.318		0.61 (0.20, 1.79)	0.369		0.54 (0.15, 1.86)	0.326	
**Tobacco smoking**	
No	1.04 (0.59, 1.83)	0.898	0.359	1.65 (0.91, 3.08)	0.104	0.410	1.22 (0.65, 2.35)	0.541	0.897
Yes	1.38 (1.06, 1.79)	0.016		1.26 (0.97, 1.64)	0.082		1.28 (0.98, 1.67)	0.070	
**Alcohol drinking**	
No	0.90 (0.47, 1.68)	0.751	0.028	1.44 (0.78, 2.76)	0.256	0.328	1.05 (0.57, 2.02)	0.876	0.067
Less frequent	1.11 (0.59, 2.15)	0.751		1.36 (1.04, 1.78)	0.025		1.41 (1.07, 1.85)	0.014	
Frequent	1.56 (0.87, 2.93)	0.148		1.44 (0.78, 2.76)	0.256		1.05 (0.57, 2.02)	0.876	

FIB4, fibrosis-4 index; CKM, cardiovascular-kidney-metabolic; OR, odds ratio; CI, confidence interval.

### 3.4 Sensitivity Analysis

After multiple imputation of missing values, the associations between baseline FIB-4 and CKM syndrome risk remained essentially unchanged (Supplementary Table 2). Fully adjusted ORs for the highest vs. lowest tertile were 1.36 (1.02–1.82) at 1 year, 1.49 (1.11–1.99) at 3 years, and 1.31 (0.95–1.80) at 5 years, which were all comparable with the complete-case estimates.

### 3.5 Incremental predictive value of FIB-4

**Figure 3:**
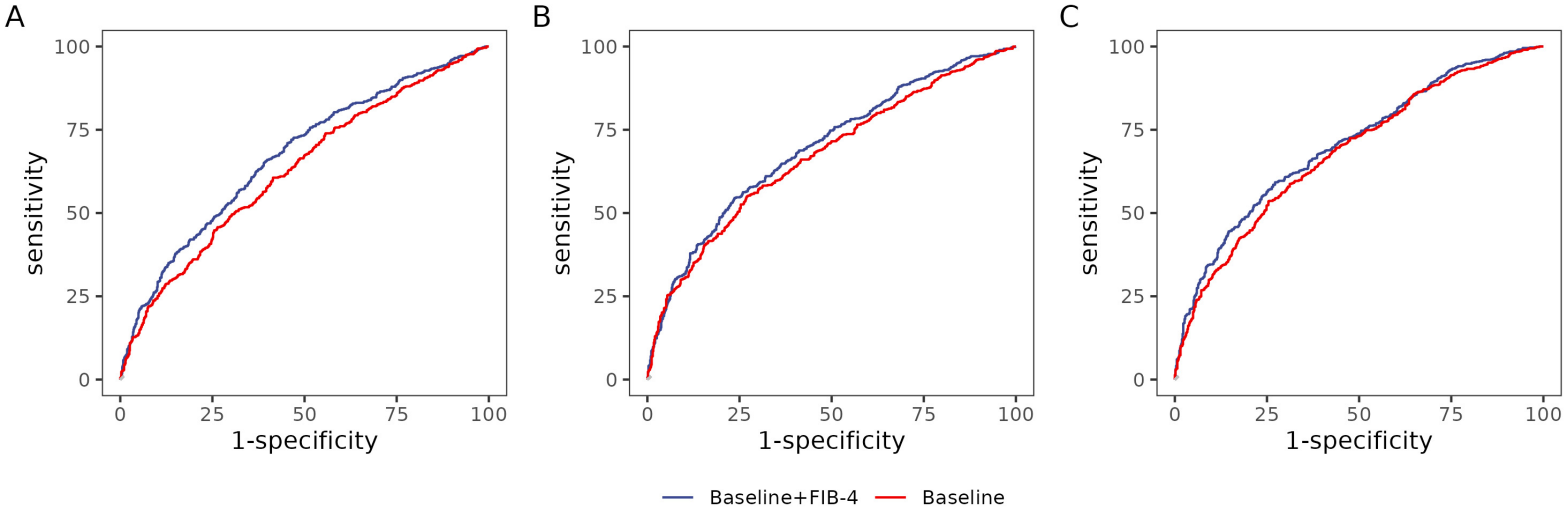
Receiver operating characteristic (ROC) curves for CKM risk prediction models at 1, 3, and 5 years. **(A–C)** correspond to 1-year, 3-year, and 5-year predictions, respectively. The diagonal line represents the reference for random chance.

Models incorporating log-FIB-4 demonstrated superior discriminative ability, with AUC values ranging from 67.2% (95% CI: 64.1–70.2%) to 70.6% (95% CI: 67.8–73.5%), compared to models without FIB-4, which yielded lower AUCs ranging from 62.9% (95% CI: 59.8–66.0%) to 68.4% (95% CI: 65.5–71.3%). For details, see Table 4 and Figure 3.

**Table 4:** AUROCs with 95% CIs (%) for CKM risk prediction models: baseline vs. baseline + FIB-4 at 1, 3, and 5 years.

**Year**	**Baseline model**	**Baseline + FIB-4 model**
1	62.9 (59.8–66.0)	67.2 (64.1–70.2)
3	67.1 (64.1–70.0)	69.5 (66.6–72.4)
5	68.4 (65.5–71.3)	70.6 (67.8–73.5)

Baseline model included age, sex, smoking, alcohol drinking, comorbidity, body mass index, renal function, blood pressure, hepatitis, lipid profile, and drug use.

AUROC, area under the receiver operating characteristic curves; FIB4, fibrosis-4 index; CKM, cardiovascular-kidney-metabolic; CI, confidence interval.

## 4 Discussion

In this retrospective cohort of 1,633 participants, elevated baseline FIB-4 predicted incident CKM syndrome at 1- and 3-year follow-up, with each one-log-unit increase in FIB-4 corresponding to a 31–32% higher risk of developing CKM syndrome. At 5 years, the association remained directionally consistent, though of borderline statistical significance (OR 1.27, 95% CI 0.99–1.63). Participants in the highest tertile manifested a 45–75% higher risk than individuals in the lowest tertile across all time points.

The spline analyses showed temporal differences in the FIB-4–CKM association, with non-linearity at 1 year transitioning to more linear patterns at 3 and 5 years. This may reflect short-term amplification of risk by acute metabolic stress or subclinical inflammation in those with moderately elevated FIB-4, whereas cumulative liver fibrosis exerts a more gradual, dose-dependent effect over longer follow-up. Earlier clinical detection in higher-risk individuals could also contribute to these temporal differences.

Our data expand FIB-4's clinical role beyond that outlined in the 2023 AHA Presidential Advisory on CKM health. The AHA currently recommends the adoption of FIB-4 to screen for advanced liver fibrosis in individuals with ≥2 metabolic risk factors as part of CKM assessment (20). However, our results indicate that FIB-4 functions as more than a marker of prevalent hepatic disease: it also independently predicts future onset of CKM syndrome.

To the best of our knowledge, ours was the first study in which the association between FIB-4 and incident CKM syndrome was examined. Because CKM syndrome integrates cardiovascular, metabolic, and kidney diseases, our observations aligned with prior longitudinal evidence that linked augmented FIB-4 to cardiovascular events and kidney dysfunction across diverse populations. In a large UK primary care cohort, higher FIB-4 predicted cardiovascular events and all-cause mortality in individuals with obesity or type 2 diabetes, and demonstrated prognostic utility beyond hepatic outcomes (18). In an Asian population similar to ours, a retrospective cohort study of Japanese men undergoing routine health check-ups revealed that higher FIB-4 levels were linked to the development of CKD and a greater decline in estimated glomerular filtration rate over 5 years, even among metabolically healthy individuals who were non-obese, normotensive, or who did not smoke (21).

Although FIB-4 has been implemented as a prognostic marker for various conditions (21, 22), its primary clinical validation remains anchored in the assessment of hepatic fibrosis (22), especially in metabolic dysfunction-associated steatotic liver disease (MASLD). This suggests that the predictive value of FIB-4 for incident CKM syndrome likely stems from overlapping mechanisms between MASLD and CKM, including insulin resistance, dyslipidemia, and chronic inflammation (1, 23–25). Central to this bidirectional relationship is the liver's dual role in MASLD: it serves as both a metabolic target of systemic dysfunction and an active driver of cardiometabolic disease. As a metabolic target, the liver is subject to injury from insulin resistance, lipid overload, and inflammatory mediators; and this results in steatosis, inflammation, and fibrosis (26). As an active driver, the liver secretes hepatokines (e.g., fetuin-A, fibroblast growth factor 21, selenoprotein *P*, and angiopoietin-like proteins) that regulate peripheral insulin sensitivity, lipoprotein metabolism, endothelial function, and systemic inflammation (27, 28). Dysregulated hepatokine secretion, coupled with hepatic overproduction of very-low-density lipoprotein and pro-inflammatory cytokines [e.g., tumor necrosis factor-alpha (TNF-α) and interleukin-6 (IL-6)], thus directly promotes disease progression (29–31). Reflecting the liver's active contribution to CKM, new definitions such as cardio–renal–diabetes–liver–metabolic syndrome (CARDIAL-MS) (32) and cardiovascular–renal–hepatic–metabolic (CRHM) syndrome (8) now explicitly incorporate hepatic status. Our results support this conceptual shift and underscore the importance of assessing liver fibrosis burden in the stratification of CKM risk.

Each FIB-4 component reflects pathways central to CKM. When aminotransferases, especially AST, are elevated, they signal liver cell and mitochondrial damage that can lead to metabolic cardiomyopathy (33, 34). This damage creates an inflammatory environment that promotes fetuin-A secretion, which, in turn, hampers insulin signaling (35, 36) and promotes atherosclerosis (37). Interestingly, this same environment triggers the production of fibroblast growth factor 21 as a natural corrective response, although receptor resistance reduces its effectiveness, as seen with insulin resistance (38). A low platelet count indicates portal hypertension caused by fibrosis, which disrupts blood flow and leads to kidney congestion (39, 40), while also reflecting ongoing systemic inflammation. Age captures the accumulated metabolic and vascular stress over time. FIB-4, therefore, integrates indicators of acute injury, structural changes, inflammation, and long-term effects, which explains its ability to predict CKM syndrome beyond traditional risk markers.

There were several limitations. Our observational design precludes causal inference; although FIB-4 temporally preceded CKM diagnosis, it should be interpreted as a risk predictor rather than a causal driver. The cohort comprised individuals undergoing routine health check-ups in a single region of Anhui, China. The high exclusion rate (95%) and our inability to compare baseline characteristics of excluded participants introduced selection bias and limited generalizability to populations with incomplete health records, lower health engagement, or different geographic and ethnic backgrounds. We used FIB-4 as a non-invasive surrogate for hepatic fibrosis rather than ultrasonography or biopsy. Although widely validated, FIB-4 is influenced by age and platelet count, which may reduce its specificity for detecting fibrosis in general populations compared to specialized hepatology settings. China-PAR was not recalibrated for our cohort, potentially affecting the precision of risk estimates. Additionally, we applied the 20% threshold per AHA guidelines rather than the 10% threshold recommended in Chinese guidelines, potentially underestimating Stage 3 CKM prevalence. Despite adjustment for major traditional risk factors, residual confounding from unmeasured variables, particularly dietary patterns, physical activity, and genetic susceptibility, remains possible. External validation in geographically and ethnically diverse, community-based populations is needed before broader clinical implementation.

## 5 Conclusion

Elevated FIB-4 was associated with incident CKM syndrome at 1 and 3 years, with suggestive evidence of a similar association at 5 years in our cohort of 1,633 participants. These data position hepatic fibrosis burden as a clinically relevant component of CKM risk and are consistent with emerging frameworks that integrate liver health into multi-organ cardiometabolic assessments. Because FIB-4 requires only routine laboratory data (age, aminotransferases, platelets), it offers a pragmatic, no-cost approach to CKM risk screening in diverse clinical settings; future work should establish optimal cutoffs and evaluate whether FIB-4-guided management improves clinical outcomes.

## Data Availability

The original contributions presented in the study are included in the article/Supplementary material, further inquiries can be directed to the corresponding authors.
