# Supplementary material for: Association of the fibrosis-4 index with early-stage cardiovascular-kidney-metabolic syndrome in a longitudinal community-based cohort
Source: Front Public Health. 2026 Mar 18;14:1793972. doi: 10.3389/fpubh.2026.1793972 (PMC13038536; doi:10.3389/fpubh.2026.1793972)
Supplement: Supplementary file 1 [file Table_1.docx]

TABLE OF CONTENT

[Supplementary Table 1. Description of the missing data 2](#_Toc21447)

[Supplementary Table 2. Associations of baseline FIB4 level with 1-, 3-, and 5-year risk of CKM syndrome across different modeling strategies in multiple imputed datasets. 3](#_Toc3312)

[Supplementary Figure 1. Fibrosis-4 index is log-transformed to approximate a normal distribution. 5](#_Toc29811)

[Supplementary Figure 2. A directed acyclic graph illustrates the potential confounders of the association between FIB-4 index and risk of CKM syndrome. 5](#_Toc23269)

| **Supplementary Table 1**. Description of the missing data | | | | | | |
| --- | --- | --- | --- | --- | --- | --- |
| Variable | Number | Proportion, % |  | Variable | Number | Proportion, % |
| Fib-4 | 0 | 0.00 |  | Sex | 0 | 0.00 |
| Fasting glucose | 6 | 0.37 |  | Age | 0 | 0.00 |
| Triglycerides | 87 | 5.33 |  | Smoking | 113 | 6.92 |
| Total cholesterol | 87 | 5.33 |  | Drinking | 112 | 6.86 |
| Low-density lipoprotein | 87 | 5.33 |  | Height | 262 | 16.04 |
| High-density lipoprotein | 87 | 5.33 |  | Weight | 262 | 16.04 |
| Creatinine | 69 | 4.23 |  | Body mass index | 261 | 15.98 |
| Blood urea nitrogen | 69 | 4.23 |  | Diastolic blood pressure | 210 | 12.86 |
| Albumin | 0 | 0.00 |  | Systolic blood pressure | 209 | 12.80 |
| Globulin | 0 | 0.00 |  | White blood cell | 746 | 45.68 |
| Total protein | 0 | 0.00 |  | Monocyte percentage | 0 | 0.00 |
| Gamma-glutamyl transferase | 258 | 15.80 |  | Neutrophil percentage | 0 | 0.00 |
| Aspartate aminotransferase | 0 | 0.00 |  | Basophil percentage | 0 | 0.00 |
| Alanine aminotransferase | 0 | 0.00 |  | Lymphocyte percentage | 0 | 0.00 |
| PH | 124 | 7.59 |  | Eosinophil percentage | 0 | 0.00 |
| Total bilirubin | 0 | 0.00 |  | Cystatin C | 801 | 49.05 |
| Direct bilirubin | 258 | 15.80 |  | Uric acid | 70 | 4.29 |
| platelet | 0 | 0.00 |  | Red blood cell | 0 | 0.00 |
| Plateletcrit | 1 | 0.06 |  | Hemoglobin | 0 | 0.00 |
| Mean platelet volume | 1 | 0.06 |  | Red-cell distribution width | 0 | 0.00 |
| Platelet distribution width | 1 | 0.06 |  | Hematocrit | 0 | 0.00 |

| **Supplementary Table 2**. Associations of baseline FIB4 level with 1-, 3-, and 5-year risk of CKM syndrome across different modeling strategies in multiple imputed datasets. | | | | | | | | | |
| --- | --- | --- | --- | --- | --- | --- | --- | --- | --- |
|  | Model 1 | | | Model 2 | | | Model 3 | | |
|  | OR (95% CI) | P-value | P for trend | OR (95% CI) | P-value | P for trend | OR (95% CI) | P-value | P for trend |
| 1-year risk of CKM syndrome | | | |  |  |  |  |  |  |
| per one log-unit | 1.61 (1.34, 1.92) | <0.001 | — | 1.29 (1.05, 1.58) | 0.014 | — | 1.27 (1.03, 1.57) | 0.025 | — |
| Tertile 1 | Reference |  | <0.001 | Reference |  | 0.031 | Reference |  | 0.066 |
| Tertile 2 | 1.09 (0.87, 1.38) | 0.451 |  | 1.05 (0.83, 1.32) | 0.696 |  | 1.01 (0.79, 1.29) | 0.926 |  |
| Tertile 3 | 1.84 (1.43, 2.36) | <0.001 |  | 1.40 (1.06, 1.85) | 0.018 |  | 1.36 (1.02, 1.82) | 0.038 |  |
| 3-year risk of CKM syndrome | |  |  |  |  |  |  |  |  |
| per one log-unit | 1.67 (1.40, 1.99) | <0.001 | — | 1.35 (1.10, 1.65) | 0.003 | — | 1.29 (1.05, 1.60) | 0.017 | — |
| Tertile 1 | Reference |  | <0.001 | Reference |  | 0.002 | Reference |  | 0.011 |
| Tertile 2 | 1.25 (1.00, 1.56) | 0.051 |  | 1.18 (0.94, 1.49) | 0.147 |  | 1.11 (0.87, 1.41) | 0.389 |  |
| Tertile 3 | 2.04 (1.58, 2.63) | <0.001 |  | 1.56 (1.18, 2.06) | 0.002 |  | 1.49 (1.11, 1.99) | 0.008 |  |
| 5-year risk of CKM syndrome | |  |  |  |  |  |  |  |  |
| per one log-unit | 1.69 (1.41, 2.02) | <0.001 | — | 1.31 (1.07, 1.62) | 0.009 | — | 1.28 (1.02, 1.59) | 0.029 | — |
| Tertile 1 | Reference |  | <0.001 | Reference |  | 0.019 | Reference |  | 0.058 |
| Tertile 2 | 1.36 (1.09, 1.70) | 0.007 |  | 1.27 (1.01, 1.59) | 0.043 |  | 1.23 (0.97, 1.56) | 0.094 |  |
| Tertile 3 | 1.92 (1.47, 2.50) | <0.001 |  | 1.36 (1.02, 1.83) | 0.039 |  | 1.31 (0.95, 1.80) | 0.097 |  |
| Abbreviations: FIB4, fibrosis-4 index; CKM, cardiovascular-kidney-metabolic; OR, odds ratio; CI, confidence interval.  Model 1 is the crude model. Model 2 adjusts age, sex, smoking, alcohol drinking, and comorbidity. Model 3 adjusts age, sex, smoking, alcohol drinking, comorbidity, body mass index, renal function, blood pressure, hepatitis, lipid profile, and drug use. | | | | | | | | | |


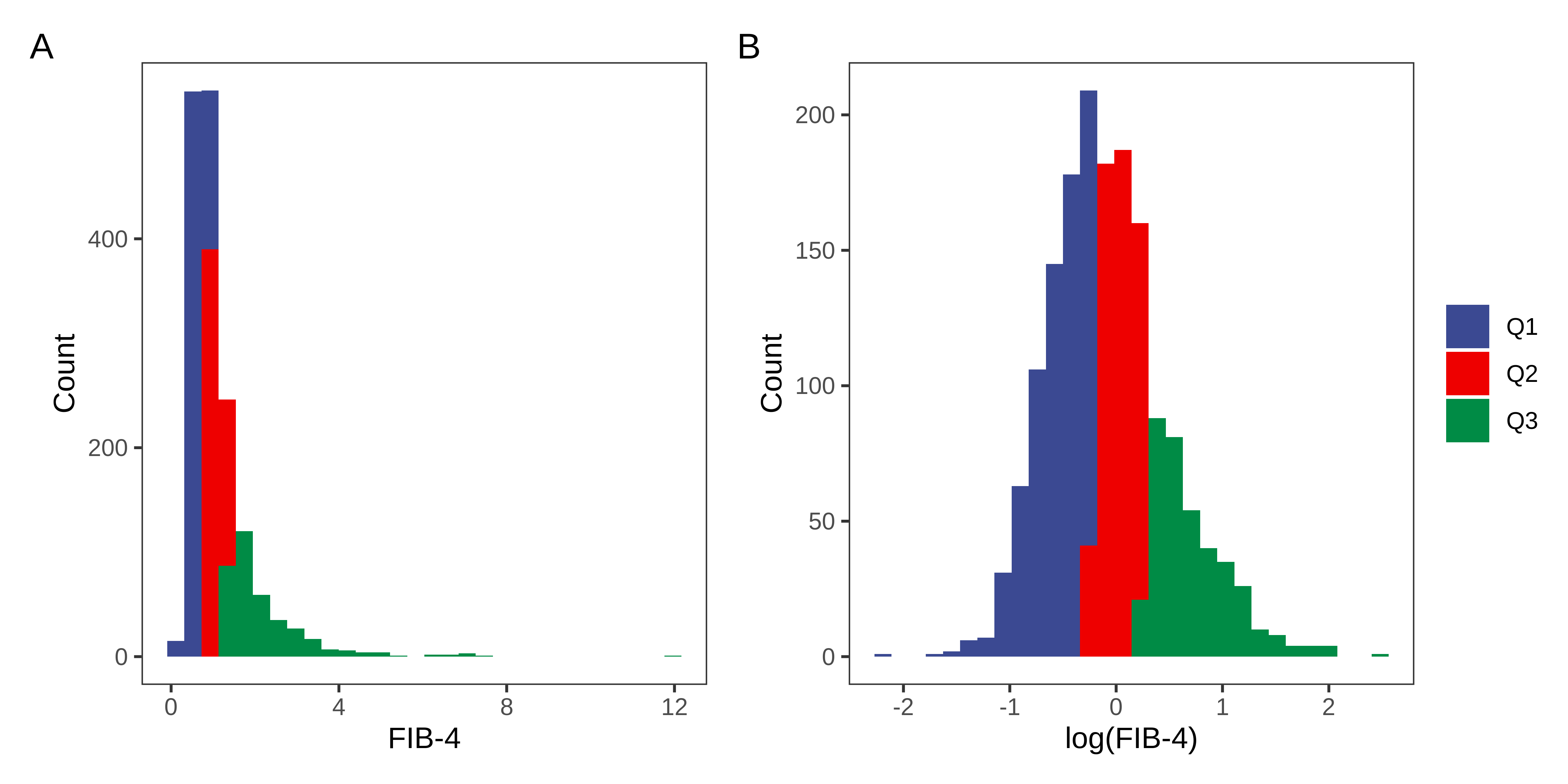


# **Supplementary Figure 1**. Fibrosis-4 index is log-transformed to approximate a normal distribution.


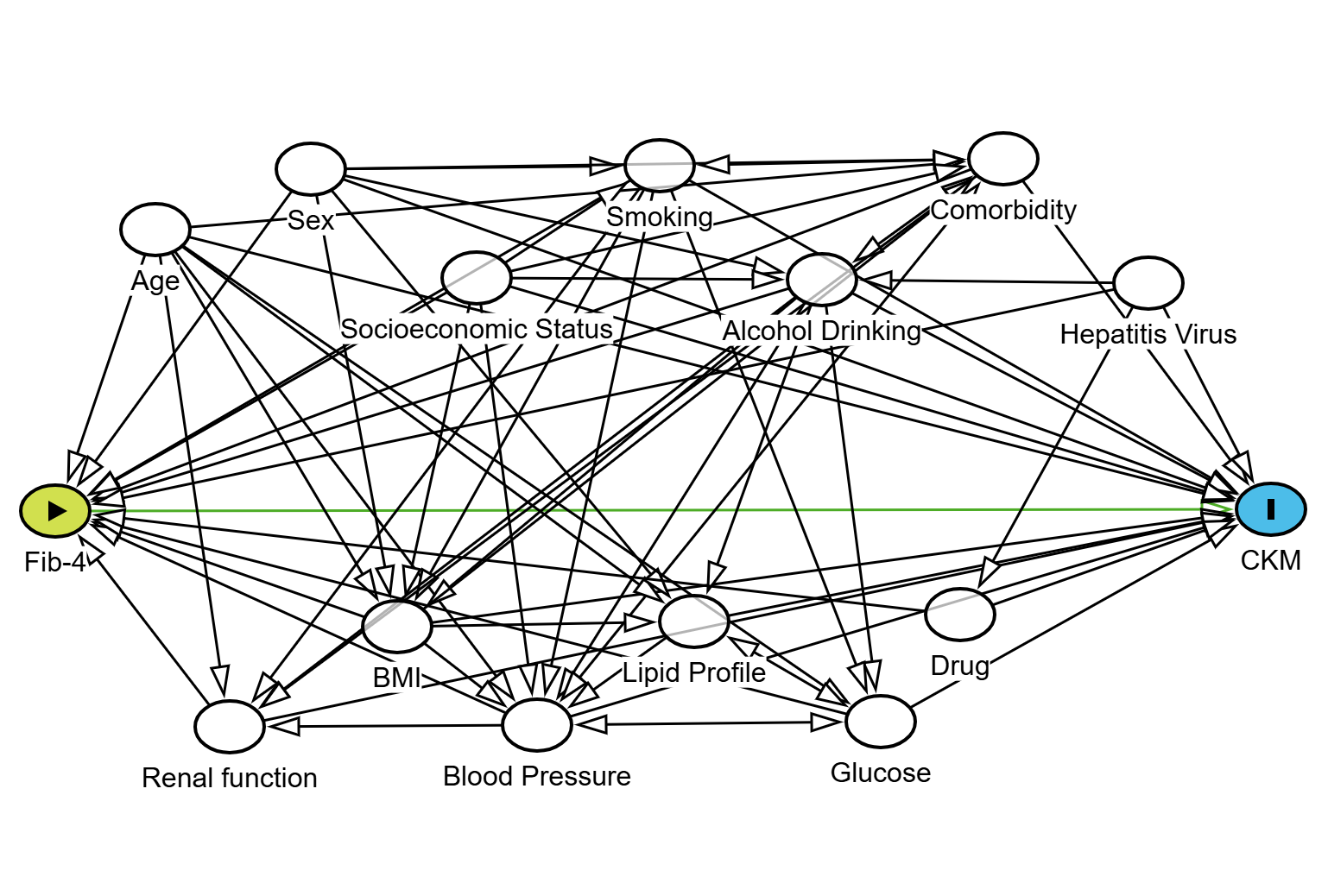


**Supplementary Figure 2**. A directed acyclic graph illustrates the potential confounders of the association between FIB-4 index and risk of CKM syndrome. Abbreviations: Fib-4, fibrosis-4 index; BMI, body mass index; CKM, cardiovascular-kidney-metabolic
